# Epimorphin expression in interstitial pneumonia

**DOI:** 10.1186/1465-9921-6-6

**Published:** 2005-01-16

**Authors:** Yasuhiro Terasaki, Yuh Fukuda, Moritaka Suga, Naoki Ikeguchi, Motohiro Takeya

**Affiliations:** 1 Department of Cell Pathology, Postgraduate School of Medicine, Kumamoto University, Kumamoto, Japan; 2 Department of Respiratory Medicine, Postgraduate School of Medicine, Kumamoto University, Kumamoto, Japan; 3 Department of Analytic Human Pathology, Nippon Medical School, Tokyo, Japan; 4 Osaka R&D Laboratory (Yokohama-lab), Sumitomo Electric Industries, Yokohama, Japan

**Keywords:** early fibrotic lesions, epithelium-mesenchyme interactions, MMP-2, re-epithelialization

## Abstract

Epimorphin modulates epithelial morphogenesis in embryonic mouse organs. We previously suggested that epimorphin contributes to repair of bleomycin-induced pulmonary fibrosis in mice via epithelium-mesenchyme interactions. To clarify the role of epimorphin in human lungs, we evaluated epimorphin expression and localization in normal lungs, lungs with nonspecific interstitial pneumonia (NSIP), and lungs with usual interstitial pneumonia (UIP); we also studied the effect of recombinant epimorphin on cultured human alveolar epithelial cells *in vitro*. Northern and Western blotting analyses revealed that epimorphin expression in NSIP samples were significantly higher than those in control lungs and lungs with UIP. Immunohistochemistry showed strong epimorphin expression in mesenchymal cells of early fibrotic lesions and localization of epimorphin protein on mesenchymal cells and extracellular matrix of early fibrotic lesions in the nonspecific interstitial pneumonia group. Double-labeled fluorescent images revealed expression of matrix metalloproteinase 2 in re-epithelialized cells overlying epimorphin-positive early fibrotic lesions. Immunohistochemistry and metalloproteinase activity assay demonstrated augmented expression of metalloproteinase induced by recombinant epimorphin in human alveolar epithelial cells. These findings suggest that epimorphin contributes to repair of pulmonary fibrosis in nonspecific interstitial pneumonia, perhaps partly by inducing expression of matrix metalloproteinase 2, which is an important proteolytic factor in lung remodeling.

## Introduction

Fetal development and morphogenesis of various tissues and organs such as hair follicles, teeth, salivary glands, mammary glands, kidneys, liver, pancreas, and lungs depend on epithelium-mesenchyme interactions [1,2]. Such interactions are believed to be important for the tissue regeneration necessary for wound healing in adults [3]. Pulmonary fibrosis is thought to be a result of wound healing or regeneration after lung injury [4-11]. Lung injury causes the epithelial basement membrane to be destroyed, which enables migration of interstitial cells into intra-alveolar spaces where they produce and deposit extracellular matrix (ECM). Regenerated epithelial cells then cover the surface of the intra-alveolar fibrotic area. If the injury is mild and focal and re-epithelialization has occurred, these epithelial cells appear widespread over early intraluminal fibrotic lesions and form intra-alveolar buds, which later become small collagen globules and do not contribute to alveolar structural remodeling [5-7,9-13]. For example, intra-alveolar buds, in a so-called organizing pneumonia pattern, are frequently observed in nonspecific interstitial pneumonia (NSIP); NSIP usually has a better prognosis than does usual interstitial pneumonia (UIP), which has fibroblastic foci without good re-epithelialization [8-10,13,14] as early fibrotic lesions. The re-epithelialization around early fibrotic lesions is similar to the process of fetal epithelial development [4-7], which suggests that epithelium-mesenchyme interactions may play a key role in repair of pulmonary fibrosis.

Epimorphin is a mesenchymal cell surface-associated protein that modulates epithelial morphogenesis in embryonic mouse organs including lungs and skin [15]. Epimorphin expression was found in fetal lungs and skin rudimentary cells, at the mesenchyme-epithelium interface. Epimorphin also directs epithelial morphogenetic processes in other gastrointestinal organs and mammary glands [15-21]. For example, primary cultured rat hepatocytes (an epithelial cell) were used to show that epimorphin induces formation of hepatocyte spheroids with a bile canaliculi-like structure, which maintained albumin production even without growth factors [16]. Epimorphin mediated mammary luminal morphogenesis by controlling expression of CCAAT/enhancer binding protein Î² [22], which is essential for proper mammary morphogenesis and for determination of the fate of mammary epithelial cells [23].

In cultured mammary epithelial cells, epimorphin augmented expression of matrix metalloproteinase 2 (MMP-2), an important proteinase in matrix degradation, and both epimorphin and MMP-2 were required for mammary gland morphogenesis [24]. During fetal rabbit lung development, MMP-2 and MT1-MMP (an activator of MMP-2) were detected in epithelial cells, and expression of active MMP-2 and MT1-MMP increased dramatically; MMP-2 and MT1-MMP were especially predominant during late development, in which there was an extremely wide alveolar surface, which indicated an important role for MMP-2 in alveoli formation [25]. MMP-2 was also up-regulated and activated in regenerated alveolar epithelial cells, which may lead to elongation and migration of these cells for repair of pulmonary fibrosis [26,27].

The epimorphin gene is highly conserved among mouse, rat, and human [15,28]: the 289 amino acid sequence of rat epimorphin/syntaxin 2 exhibits 86% homology to human epimorphin [29]. However, the distribution and function of epimorphin in human lungs, including during fibrosis, are unknown. To clarify the role of epimorphin in human lungs, especially in fibrosis, we evaluated epimorphin expression and localization in normal control lungs and in lungs affected by NSIP or UIP, especially in early intra-alveolar fibrotic lesions. We also used cultured human alveolar epithelial cells to assess the effect of recombinant epimorphin on MMP-2 expression.

## Materials and Methods

### Patients

All histologic slides from open or thoracoscopic lung biopsies in the files of the Department of Pathology, Kumamoto University Hospital, from 2000 to 2003, were evaluated. From among these samples, 17 patients were selected, with 8 fulfilling the histologic and clinical criteria for NSIP and 9 showing UIP. To confirm the histologic diagnosis, two pulmonary pathologists reviewed the slides; they were informed only of the age and sex of each patient and the presence of bilateral pulmonary disease determined by chest posteroanterior radiography and high-resolution computed tomography. All patients had had no steroid therapy before the biopsy. Normal control lung tissues were obtained from the normal areas of lungs that had been surgically removed from eight patients because of cancer. Table 1 shows the characteristics of the nine patients with UIP, eight patients with NSIP, and eight normal control subjects. In addition to the histologic diagnosis, we confirmed that the clinical data, pulmonary function test results, bronchoalveolar lavage findings, chest radiographic findings, and follow-up information obtained from the patients were consistent with the diagnoses. Diagnostic criteria of the American Thoracic Society/European Respiratory Society consensus classification system were applied [30]. We also verified that none of the patients with UIP had a connective tissue disease. In the group of patients with NSIP, two with SjÃ¶gren's syndrome and one with rheumatoid arthritis were included. In all other cases, no etiologic agent was found. These tissue samples were used for microscopic immunohistochemistry, Western blotting, and Northern blotting assays. The procedures used in this study were in accordance with those recommended by the regional ethical committee on human experimentation.

### Northern Blotting Studies

Human epimorphin DNA fragments were isolated by reverse transcriptase-polymerase chain reaction. Strand cDNA was synthesized with random primers from human lung total RNA. Polymerase chain reaction assays were carried out at 95Â°C for 1 minute, 64Â°C for 1 minute, and 72Â°C for 1 minute for 35 cycles. The following primer pairs were used: human epimorphin forward 5'-GGA ACC GGA CTT CAG TGG ATC-3' and reverse 5'-CAGC CAA TGA TTA GAG CCA GGA-3'. Polymerase chain reaction products were subcloned into the *Nco*I and *Spe*I sites of the pGEM-T Vector (Promega, Madison, WI), and authenticity was confirmed by sequencing. Total cellular RNA was isolated from lung tissues from each case by using the acid guanidinium-isothiocyanate-phenol-chloroform method. The RNA samples (10 Î¼g/lane) were fractionated by electrophoresis on 1% agarose-formaldehyde gels under denaturing conditions and were transferred to Nytran by capillary action. The blots were then probed with the 339-bp *Nco*I/*Spe*I fragment of the human epimorphin cDNA labeled with [^32^P]dCTP (3000 Ci/mmol) by means of the Nick Translation System kit (GIBCO/BRL, Grand Island, NY). After hybridization, blots were washed and were processed by autoradiography. Washed blots were analyzed by using a Fujix BAS2000 Bio-Imaging analyzer system (BASTM, Fuji Photo Film Co., Ltd., Tokyo, Japan) and were visualized on Kodak X-OMAT AR film (Eastman Kodak, Rochester, NY). Signal intensity was quantified in digital images via BASTM analysis (FUJIX BAStation, Fuji Photo Film Co., Ltd.). To control for differences in gel loading, for each sample the RNA hybridized with the epimorphin probe was normalized to the expression of Î²-actin mRNA in the same sample.

### Western Blotting Studies

Although the monoclonal antibody to mouse epimorphin (MC-1) has been reported to cross-react with human epimorphin [31,32], we performed Western blotting to confirm whether the anti-epimorphin antibody (MC-1, a gift from Dr. Hirai, Osaka R&D Laboratory (Yokohama-lab), Sumitomo Electric Industries, Yokohama, Japan) cross-reacted with the human epimorphin in our lung samples and to make comparisons of epimorphin peptide expression between normal and fibrotic groups. For immunoblotting, lung tissues from each case were dissolved in sample buffer containing 2% sodium dodecyl sulfate in 8 M urea and were kept at 4Â°C overnight; the same protein concentration (30 Î¼g) in samples from each lung extract was ensured by using Protein Assay (Bio-Rad Laboratories, Hercules, CA). Lung tissues from normal adult mice were also dissolved in sample buffer as above and served as positive controls. Those samples were subjected to sodium dodecyl sulfate-polyacrylamide gel electrophoresis according to the method of Laemmli (1970), and proteins in the gels were transferred onto nitrocellulose filters and were incubated sequentially with 5% skim milk in 0.5% Tween 20 overnight. Blots were then incubated at 4Â°C overnight with 10 Î¼g/ml rat anti-mouse epimorphin primary monoclonal antibody MC-1 used at a dilution of 1:1000 in 1% bovine serum albumin. Samples were then stained by incubation with horseradish peroxidase-conjugated anti-rat goat IgG F(ab')_2 _antibody (Biosource International Inc., Camarillo, CA) used at a dilution of 1:1000 in Tris-buffered saline with 0.05% Tween 20 for 2 hours at room temperature. The bound antibody was detected by using the enhanced chemiluminescence method (Amersham Pharmacia Biotech UK Limited, Little Chalfont, Buckinghamshire, England) according to the manufacturer's protocol.

### Light Microscopic Immunohistochemistry

A portion of each lung specimen was fixed immediately in a solution of 4% paraformaldehyde in 0.1 M phosphate buffer (pH 7.4), after which samples were sequentially washed for 4 hours in 10% sucrose in 0.01 M phosphate-buffered saline (pH 7.4, 4Â°C), 4 hours in 20% sucrose in phosphate-buffered saline, and overnight in 30% sucrose in phosphate-buffered saline. They were then snap-frozen in OCT embedding medium and stored at -80Â°C.

Frozen tissues were cut into 4-Î¼m sections, which were incubated for 30 minutes with a biotin blocking system (Dako Corporation, Carpinteria, CA) and for 30 minutes with 0.3% hydrogen peroxide in methanol to eliminate endogenous peroxidase activity. After treatment with normal goat serum, tissues were incubated overnight at 4Â°C with 10 Î¼g/ml rat anti-mouse monoclonal epimorphin antibody and were then incubated for 1.5 hours at 37Â°C with 1 Î¼g/ml biotinylated goat anti-rat IgG (ZYMED, San Francisco, CA) and streptavidin-biotin-horseradish peroxidase complex (Dakopatts, Glostrup, Denmark). Bound antibody was visualized after incubation for 10 minutes in a Coplin jar with 100 ml of Tris-HCl buffer (pH 7.6) containing 20 mg of diaminobenzidine and 17 ml of 30% H_2_O_2_. Counterstaining was with Mayer's hematoxylin. Lung tissue of normal adult mice was stained as a positive control. Nonspecific labeling of primary antibody was evaluated with normal rat serum. Each tissue sample was also stained for keratin (rabbit anti-bovine antibody raised against the 58-, 56-, and 52-kd subunits of muzzle epidermal keratin; Dakopatts, Santa Barbara, CA), Î±-smooth muscle actin (mouse anti-human Î±-smooth muscle actin monoclonal antibody; Dakopatts, Santa Barbara, CA), and MMP-2 (mouse anti-human MMP-2 monoclonal antibody; clone 42-5D11, IgG1 isotype, purified antibody; Daiichi Fine Chemical Co., Ltd., Takaoka, Japan). Serial sections were also stained with hematoxylin-eosin (H&E) and Alcian blue-periodic acid-Schiff (AB-PAS).

### Confocal Microscopy

To localize epimorphin as precisely as possible, epithelial and stromal cells were double labeled by means of immunofluorescent probes, and the distribution of epimorphin was compared with that of keratin and vimentin (mouse monoclonal anti-vimentin [V9] antibody; Dakopatts, Glostrup, Denmark). Briefly, after sections were exposed to primary antibodies, they were exposed to secondary antibodies: for the first analysis: fluorescein isothiocyanate-conjugated goat anti-rat IgG (American Qualex, San Clemente, CA) or Texas Red-conjugated goat anti-rabbit IgG (Molecular Probes, Inc., Eugene, OR); for the second analysis: fluorescein isothiocyanate-conjugated goat anti-rat IgG (American Qualex) or Texas Red-conjugated goat anti-mouse IgG (Molecular Probes). The nuclei were counterstained with 4,6-diamidino-2-phenylindole dihydrochloride (Vector Laboratories, Inc., Burlingame, CA). Specimens were examined under a confocal laser scanning microscope (TCS-SP; Leica Lasertechnik, Heidelberg, Germany), based on an upright microscope (DMRB, Leica Lasertechnik) equipped with a krypton/argon laser [33]. The excitation wavelengths for fluorescein isothiocyanate and Texas Red were 498 nm and 568 nm, respectively. Green fluorescein isothiocyanate emission was selected and recorded by using a 500- to 550-nm bandpass filter; red Texas Red emission was selected and recorded by using a 581- to 631-nm bandpass filter. In addition, 4,6-diamidino-2-phenylindole dihydrochloride was excited at 350-nm by using a UV laser, and its blue emission was recorded via a 401- to 551-nm bandpass filter.

### Localization of MMP-2 and Epimorphin in Lungs with NSIP

To compare the precise localization of MMP-2 and epimorphin in NSIP, a confocal microscope was used for immunohistochemical study of lung tissues from patients with NSIP, by means of the same technical immunohistochemical staining and analysis method as that described previously for epimorphin and keratin detection [34]. Mouse anti-human MMP-2 monoclonal antibody and rat anti-mouse monoclonal epimorphin antibody served as primary antibodies. Secondary antibodies for double-labeling immunohistochemistry studies via confocal microscopy were goat anti-mouse IgG antibody (Alexa Fluor 546; Molecular Probes) and goat anti-rat IgG antibody (Alexa Fluor 488; Molecular Probes).

### Cell Culture and Treatment Conditions

Human lung-derived alveolar epithelial cells (A549), an epithelial cell line derived from lung adenocarcinoma (Cell Resource Center for Biomedical Research, Institute of Development, Aging and Cancer, Tohoku University), were cultured in RPMI (GIBCO/BRL) supplemented with 10% fetal bovine serum, 2 mM l-glutamine, 50 U/ml penicillin, and 50 Î¼g/ml streptomycin. All cells were maintained at 37Â°C in a humidified incubator containing 5% CO_2 _and 95% air.

### Detection of MMP-2 Expression in A549 Cells

Four-well chamber slides (Lab-Tek II, Nalge Nunc International, Naperville, IL) were coated with human recombinant epimorphin fragment (H12; 20 Î¼g/well, dissolved in 1.5 mM HCl; a gift from Dr. Hirai), negative control solution prepared from non-transfected *Escherichia coli *strain BL21 (BL; 20 Î¼g/well, dissolved in 1.5 mM HCl; a gift from Dr. Hirai), or Type IV collagen from human placenta collagen (20 Î¼g/well, dissolved in 0.4% glacial acetic acid; Sigma Chemical Co., St. Louis, MO), after which samples were dried at room temperature. After A549 cells were washed two times with phosphate-buffered saline, the cells (5 Ã— 10^5^) were suspended in RPMI medium with 2% fetal calf serum and were incubated at 37Â°C on the chamber slides coated with recombinant epimorphin, BL, or Type IV collagen. After incubation for 9 h, culture dishes were placed on ice, medium from each dish was collected, and cells were washed twice with phosphate-buffered saline. Cells were fixed in 4% paraformaldehyde in phosphate-buffered saline for 5 minutes at -20Â°C and then were washed in phosphate-buffered saline and permeabilized in acetone for 5 minutes at -20Â°C. Cells were again washed in phosphate-buffered saline and incubated with a blocking solution containing 1% bovine serum albumin/phosphate-buffered saline for 1 hour at room temperature. Immunohistochemistry was performed via the same technical method described above for epimorphin detection. The primary antibody was 5 Î¼g/ml mouse anti-human MMP-2 monoclonal antibody, and the secondary antibody was horseradish peroxidase-conjugated goat anti-mouse IgG antibody (Amersham Pharmacia Biotech UK Limited).

The A549 culture supernatant collected after incubation for 9 hours on the chamber slides coated with recombinant epimorphin, BL, and Type IV collagen was used to determine the amount of MMP-2 activity by means of the MMP-2 Biotrack Activity Assay System (Amersham Pharmacia Biotech UK Limited), according to the manufacturer's instructions. All samples were assayed in triplicate, and assays were repeated three times.

### Statistical Analysis

Densitometric analysis of the bands of Northern and Western blotting was performed with Macintosh G4 computer (Apple Japan, Inc., Tokyo, Japan) using high-resolution scanner and NIH image software (version 1.62, National Institutes of Health, Bethesda, Maryland). The data are expressed as a ratio of a standard normal control subject to each case's band density units (arbitrary units) and reported as mean Â± standard error of the mean (SEM). Statistical significance was established using the one-way analysis of variance test followed by Tukey-Kramer multiple intergroup comparison test. Probabilities less than 0.05 were considered significant.

## Results

### Expression of Epimorphin mRNA in Normal Human Lungs and Lungs of Patients with NSIP or UIP

**Figure 1:**
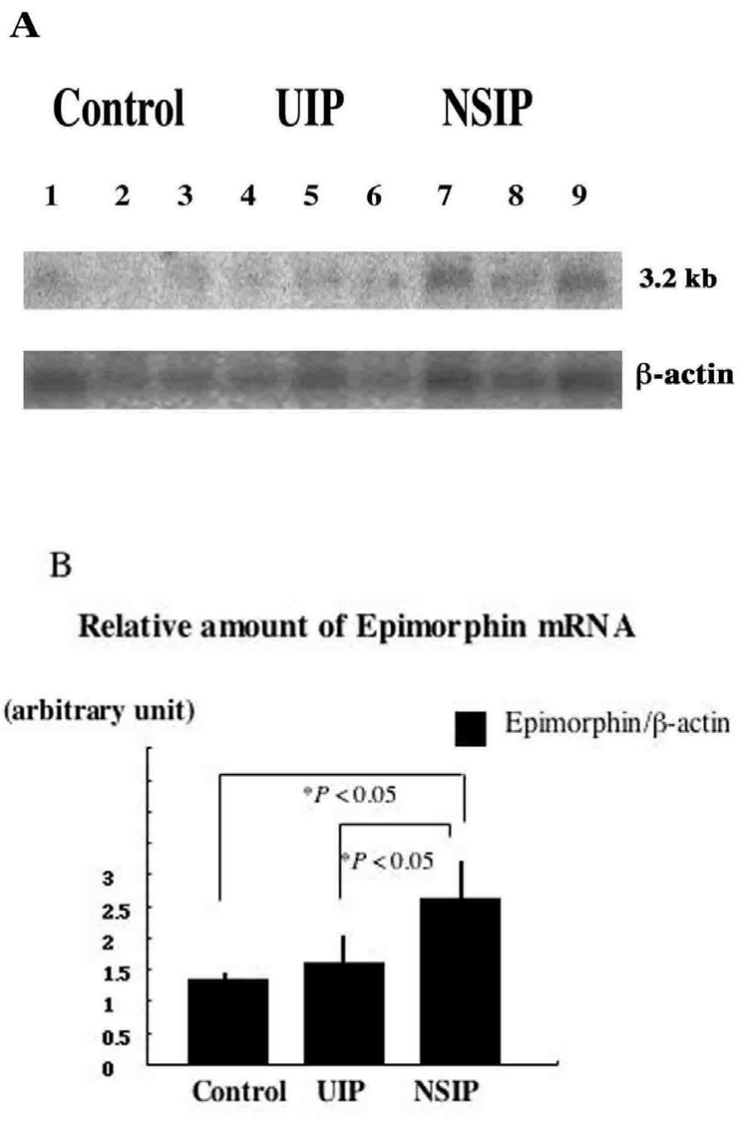
(A) Representative Northern blots of epimorphin mRNA and Î²-actin mRNA. Epimorphin mRNA (3.2-kb) was expressed in samples of normal lung (Control: *lanes 1â€“3*) and lungs from patients with UIP (*lanes 4â€“6*) and patients with NSIP (lanes 7â€“9) (*upper blot*). To confirm that the blots reflected samples of equal size, they were reprobed for expression of Î²-actin mRNA (*lower blot*). (B) The data are expressed as a ratio of epimorphin to Î²-actin band density units (arbitrary units) and reported as mean Â± SEM measured by means of NIH Image analysis. In NSIP samples, the density of epimorphin expression was significantly higher than that in control and UIP samples (*P < 0.05).

Expression of 3.2-kb epimorphin mRNA was revealed in all lung samples from patients with NSIP, UIP and normal control subjects by Northern blotting (Figure 1A). To confirm that the blots reflected samples of equal size, they were reprobed for expression of Î²-actin mRNA. Compared with corresponding densities of Î²-actin, the densities of epimorphin expression in NSIP samples were significantly higher than those in control lungs and lungs with UIP, as determined by NIH Image analysis (*P *< 0.05) (Figure 1B).

### Tissue Localization of Epimorphin in Normal Human Lungs

**Figure 2:**
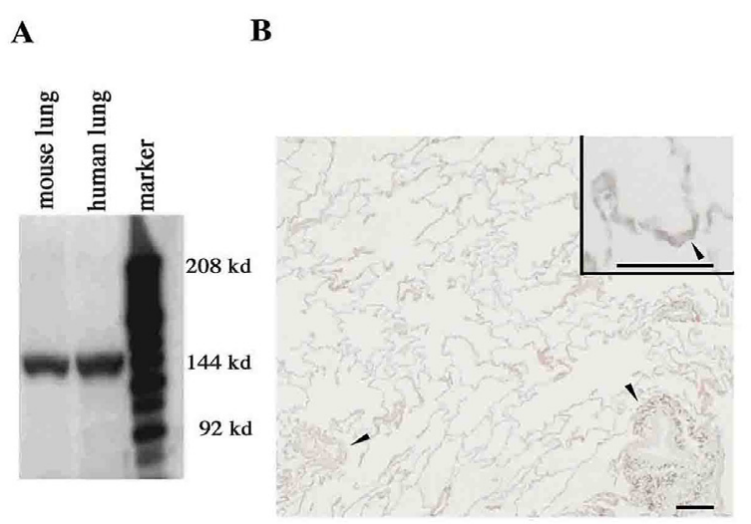
Representative Western blots for epimorphin in normal mouse and human lung samples (A), and representative epimorphin immunohistochemistry (B) in normal mouse lung. (A) Recognition of the 150-kd bands by rat anti-mouse epimorphin monoclonal antibody (MC-1) in extracted lung samples. (B) Weak epimorphin staining in vascular walls (*arrowheads*) and alveolar walls (*inset*). Scale bar = 50 Î¼m.

Western blotting showed that rat anti-mouse epimorphin monoclonal antibody (MC-1) recognized the 150-kd bands in the extracted samples from both mouse lung and normal human lung (Figure 2A), similar to results reported elsewhere [31,32]. We thus confirmed that anti-mouse epimorphin antibody demonstrated specificity and cross-reactivity with the human epimorphin of our lung samples. Normal lung tissues of the human samples had normally opened alveoli with infiltration of a few inflammatory cells. In immunohistochemical studies of the human samples, as with normal mouse lung specimens [34], epimorphin was weakly stained in vascular and alveolar walls (Figure 2B). Negative control staining with normal rat serum showed no positive findings in the serial section (data not shown).

### Amount of Epimorphin in Normal Human Lungs and Lungs of Patients with NSIP or UIP

**Figure 3:**
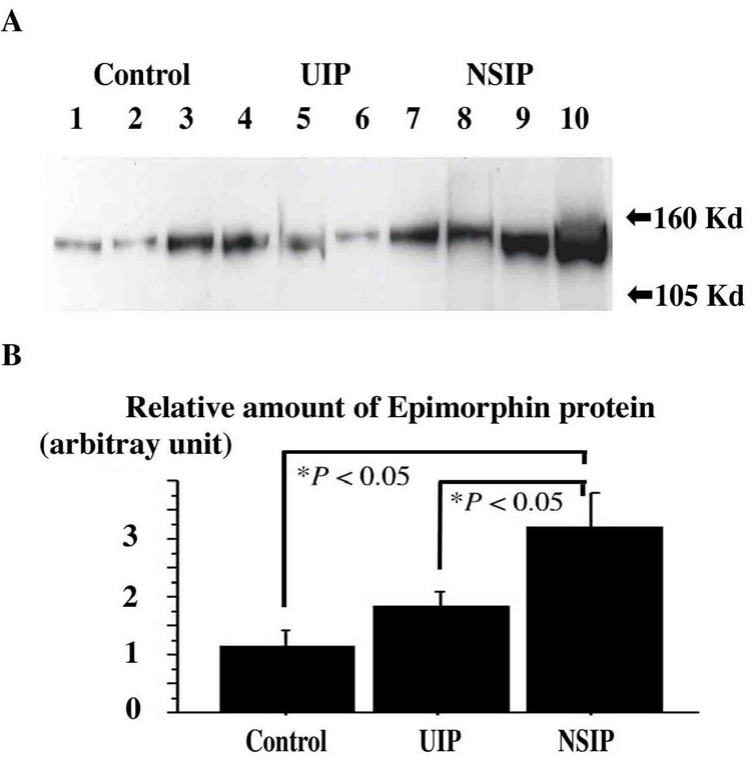
(A) Representative Western blots for epimorphin in normal control subjects and fibrotic groups. Epimorphin protein (150-kd) was expressed in samples of normal lung (Control: *lanes 1â€“3*) and lungs from patients with UIP (*lanes 4â€“7*) and patients with NSIP (*lanes 8â€“10*). (B) The data are expressed as a ratio of a standard normal control subject to each case's band density units (arbitrary units) and reported as mean Â± SEM measured by means of NIH Image analysis. In NSIP samples, the density of epimorphin protein expression was significantly higher than that in control and UIP samples (*P < 0.05)

In the lung tissue homogenates of patients with NSIP, UIP and of control subjects, Western blotting with antibody for epimorphin also recognized the 150-kd bands (Figure 3A) and the densities of the bands of patients with NSIP are relatively higher than those of UIP and control subjects by methods of NIH image analysis (p < 0.05) (Figure 3B).

### Tissue Localization of Epimorphin in NSIP and UIP by Means of Immunohistochemical Analyses

**Figure 4:**
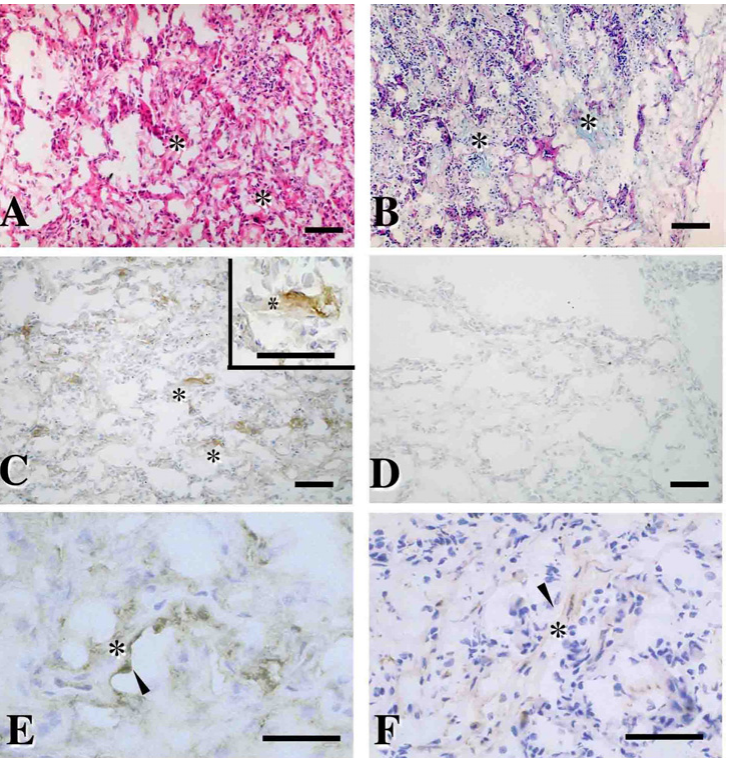
Representative histology stained with hematoxylin-eosin (H&E)(A) and Alcian blue-periodic acid-Schiff (AB-PAS)(B) and immunohistochemistry for epimorphin (C), rat normal serum as negative control for epimorphin (D), keratin (E), and Î±-smooth muscle actin (F) for all patients with NSIP. (A) Thickened alveolar walls showed edema, fibrosis, and inflammatory cell infiltration. Dense fibrosis was inconspicuous or absent. (A and B) Intra-alveolar organizing fibrotic areas were seen as pale eosinophilic fibrosis with H&E staining and as Alcian blue positive area with AB-PAS staining as early stage of fibrosis (*asterisks*). (C) Positive epimorphin immunostaining appeared in areas of early fibrosis (*inset *in C shows a close-up view; asterisks indicates early intra-alveolar fibrotic area) covered with regenerated alveolar epithelial cells (*arrowhead *in E), in addition to the alveolar walls. A few Î±-smooth muscle actin-positive cells were noted in the fibrotic areas (*arrowhead *in F). Scale bars = 60 Î¼m.

In lungs of all patients with NSIP, alveolar walls were thickened with edema, fibrosis, and inflammatory cell infiltration; the appearance was typically uniform (Figure 4A). There was slight or no dense fibrosis; intra-alveolar organizing fibrosis was seen as pale eosinophilic fibrosis with H&E staining and as Alcian blue positive area with AB-PAS staining as early stage of fibrosis (Figure 4A and 4B). Epimorphin immunostaining was strong in the early intra-alveolar fibrotic areas with a covering of regenerated alveolar epithelial cells (Figure 4C and 4E), which contained a few Î±-smooth muscle actin-positive cells (Figure 4F), in addition to immunostaining seen in vascular and alveolar walls. No positive findings was shown using normal rat serum as fist antibody for negative control (Figure 4D). The epimorphin immunohistochemical staining patterns for all eight patients with NSIP were quite similar.

**Figure 5:**
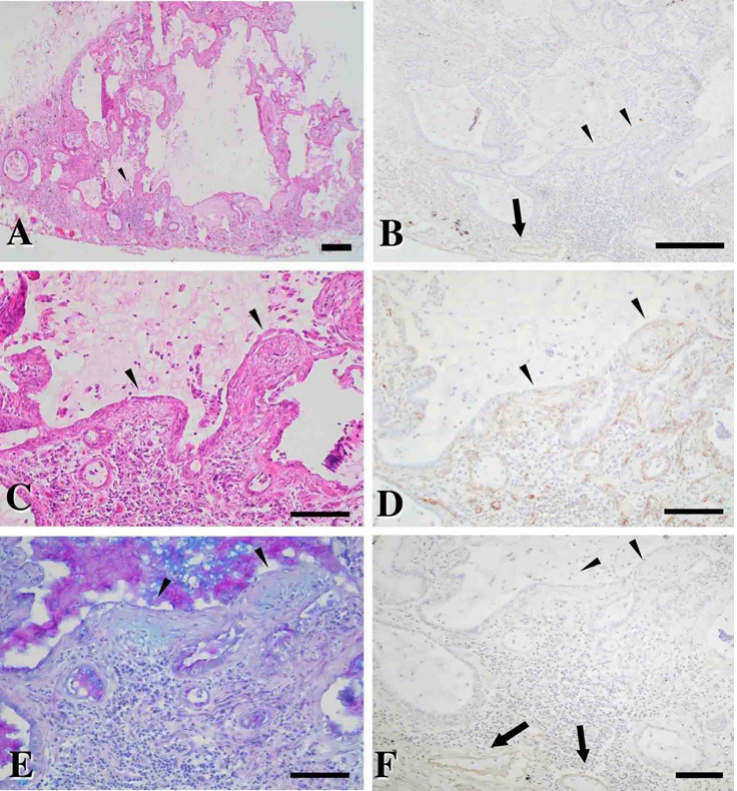
Representative histology (A, C [close-up view]; H&E staining) and immunohistochemistry for epimorphin (B, F [close-up view]) and Î±-smooth muscle actin (D [close-up view]) of scattered fibroblastic foci in early fibrotic areas as Alcian blue positive area (E; AB-PAS staining) for patients with UIP. (A and C) Honeycombing with complete architectural destruction occurred in fibrotic zones. (C and E) Arrowheads indicate scattered fibroblastic foci. In addition to clear immunostaining in the vascular and alveolar walls (*arrows*), only weak epimorphin immunostaining was seen in scattered fibroblastic foci (*arrowheads*) (B and F); many Î±-smooth muscle actin-positive cells (D) and desquamative regenerated alveolar epithelial cells (C) were found. Scale bars = A, B: 200 Î¼m; Câ€“E: 80 Î¼m.

In lungs of all patients with UIP, the fibrotic zones showed temporal heterogeneity, with dense acellular collagen and scattered fibroblastic foci with intervening nearly normal alveoli. Most fibrotic zones had honeycombing with complete destruction of the architecture (Figure 5A). Though clear immunostaining in the vascular walls, only weak epimorphin immunostaining occurred in dense fibrotic lesions and scattered fibroblastic foci, as in areas of early fibrosis (Figure 5B and 5F) with Alcian blue positive (Figure 5E). The scattered fibroblastic foci contained many Î±-smooth muscle actin-positive cells (Figure 5D) with the desquamative regenerated alveolar epithelial cells overlying these fibroblastic foci (Figure 5C).

### Tissue Localization of Epimorphin in NSIP by Means of Double-labeled Immunohistochemical Analyses

**Figure 6:**
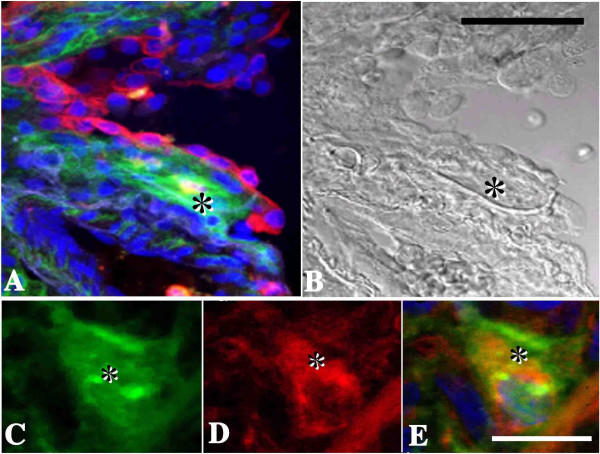
Representative double-labeled confocal fluorescent images of staining for epimorphin plus keratin (A), epimorphin (C), vimentin (D), and epimorphin plus vimentin (E) in an early fibrotic lesion from the lung of a patient with NSIP. (A) The presence of epimorphin (green) in areas of early fibrosis and the presence of keratin (red) in epithelial cells overlying the lesions were confirmed. (B) The same image shown in part A but with Nomarski optics used. (Câ€“E) Epimorphin (C, green) localized in vimentin-positive (D, red) stromal cells (yellow-orange) and in surrounding ECM in early fibrotic areas (E). Nuclei were 4,6-diamidino-2-phenylindole dihydrochloride-positive (blue). Asterisks indicate early fibrotic areas. Scale bars = B: 20 Î¼m, E: 5 Î¼m.

Double-labeled confocal fluorescent images confirmed the presence of epimorphin in early fibrotic lesions and keratin-positive epithelial cells overlying the lesions (Figure 6A and 6B). Double-labeled confocal fluorescent images also verified the localization of epimorphin in vimentin-positive stromal cells and in surrounding ECM (Figure 6C, D, and 6E) in early fibrotic lesions.

### Localization of MMP-2 and Its Relation to Epimorphin in NSIP

**Figure 7:**
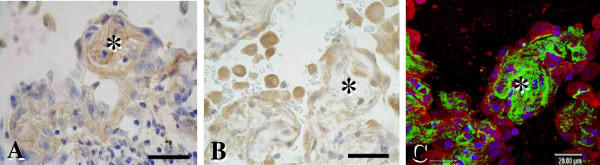
Representative images of immunostaining for epimorphin (A) and MMP-2 (B) in NSIP; the double-labeled confocal fluorescent image of in an early fibrotic lesion from the lung of a patient with NSIP was stained for epimorphin plus MMP-2 (C). (A) Epimorphin immunoreactivity was observed in early fibrotic areas, with (B) MMP-2 labeled in regenerated epithelial cells overlying the fibrotic lesion. Asterisks indicate early fibrotic areas. (C) MMP-2 (red) was strongly expressed in re-epithelialized cells overlying early fibrotic lesions with clear epimorphin expression (green). Nuclei were 4,6-diamidino-2-phenylindole dihydrochloride-positive (blue). Asterisks indicate early fibrotic areas. Scale bars = 20 Î¼m.

Areas of early fibrosis in lung tissues from patients with NSIP showed epimorphin immunoreactivity (Figure 7A), and regenerating epithelial cells overlying these lesions demonstrated MMP-2 labeling (Figure 7B). Furthermore, in tissues from patients with NSIP, double-labeled confocal fluorescent images confirmed the expression of MMP-2 (Figure 7C) in re-epithelialized cells overlying fibrotic lesions in which epimorphin was also clearly expressed.

### Increased Expression of MMP-2 Induced in A549 Cells by Epimorphin

**Figure 8:**
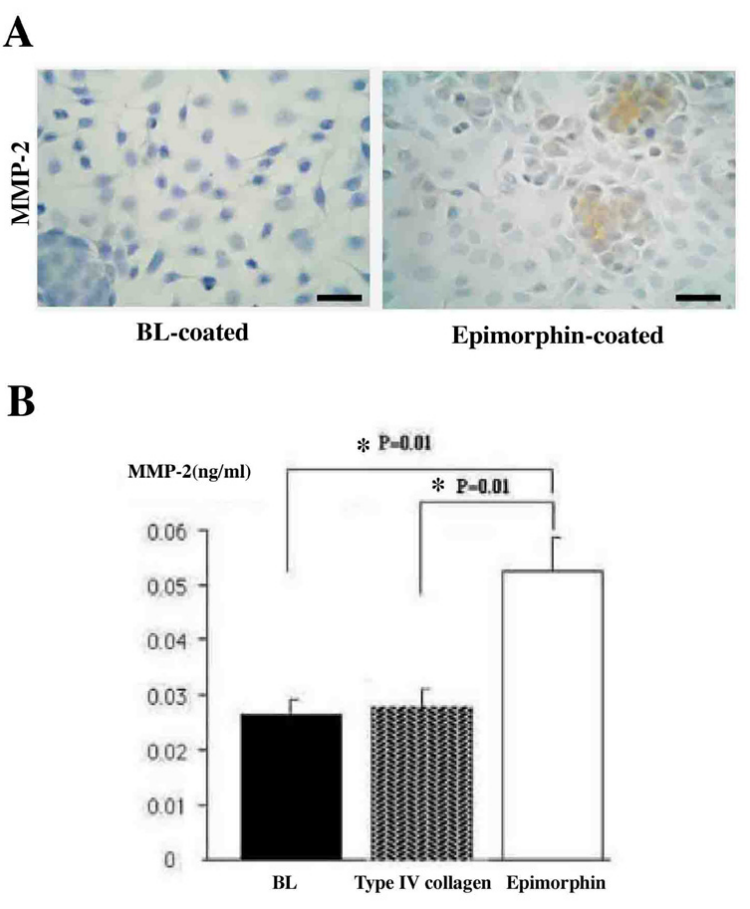
(A) Representative images of MMP-2 immunostaining in A549 cells cultured with recombinant epimorphin (*right panel*) or with BL (*left panel*) after 9 hours of incubation. In the epimorphin-coated slides, plated cells formed some monolayer cell islands in addition to loose sheets of cells, and cells in these cell islands showed strong MMP-2 immunostaining (by the immunoperoxidase method). Scale bars = 30 Î¼m. (B) Via the MMP-2 Biotrack Activity Assay System, MMP-2 activity in the supernatants of A549 cells cultured with recombinant epimorphin was higher than in supernatants of cells cultured with BL or Type IV collagen (*P *= 0.01).

Nine hours after plating of BL or Type IV collagen-coated chamber slides with cells of the human lung-derived alveolar epithelial cell line A549, the cells had spread and formed a loose sheet (Figure 8A, left panel). However, during the same time period, in recombinant epimorphin-coated chamber slides, the A549 cells formed some monolayer cell islands in addition to the loose cellular sheet, in about 50% of the chamber slide area (Figure 8A, right panel). Moreover, the immunoperoxidase method revealed strong MMP-2 immunostaining in cells of these cell islands in epimorphin-coated slides after 9 hours of incubation (Figure 8A, right panel). The MMP-2 Biotrack Activity Assay System revealed significantly higher MMP-2 activity levels in the supernatants of cultures of A549 cells with recombinant epimorphin compared with those for cultures with BL or Type IV collagen after 9 hours of incubation (Figure 8B).

## Discussion

This is the first study to examine in detail the distribution and magnitude of epimorphin expression in normal human lungs and lungs from patients with NSIP or UIP. We found clear expression of epimorphin mRNA and protein in lung samples from normal control subjects and patients with NSIP or UIP.

### Expression of Epimorphin in Normal Human Lungs

Consistent with previous reports [32], epimorphin was expressed in connective tissue components of walls of the alveoli and blood vessels in normal lungs, as determined by immunohistochemistry. Epimorphin expression was also confirmed at the mRNA and protein levels by Northern and Western blotting assays, similar to results found previously for mice [15,32]. Although the specific function of epimorphin in normal lungs is not yet known, it may serve as an epithelial and endothelial cell morphogen to maintain the cell turnover necessary for normal structure and function. Alveolar epithelial cells are replaced at regular intervals under physiologic conditions. For example, the estimated turnover time for normal mouse alveolar epithelial cells ranges from 28 to 35 days [35], and daily endothelial cell turnover is reported to be about 1% [36]. Also, in the normal adult human kidney, similar to results for normal adult mouse kidney, low levels of epimorphin mRNA and protein were detected in the glomerular mesangium and peritubular interstitium (interstitial fibroblast-like cells), as revealed by reverse transcriptase -polymerase chain reaction and immunohistochemistry [37]. These findings are consistent with our result that epimorphin is expressed in the interstitium in human control lungs, as in normal adult mouse lungs [34].

### Expression of Epimorphin in Lungs of Patients with NSIP or UIP

We found, by means of Northern blotting assays, distinct expression of epimorphin mRNA and protein in normal lungs and lung samples of patients with NSIP or UIP, with significantly higher epimorphin expression in lungs of patients with NSIP than in other lung samples. Also, serial sections and double-labeled immunohistochemistry analyses of NSIP samples demonstrated strong expression of epimorphin in mesenchymal cells situated within active intra-alveolar fibrotic lesions and the presence of epimorphin in the ECM in the vicinity of these cells. These findings are consistent with our previous report that epimorphin was synthesized by mesenchymal cells and localized to these cells and the ECM of early intra-alveolar fibrotic lesions in a murine bleomycin-induced pulmonary fibrosis model [34]. They are also consistent with a report that epimorphin was synthesized by normal human skin fibroblasts [38].

It is well known that regeneration of alveolar and bronchiolar epithelial cells is crucial for repair of pulmonary fibrosis, and the ECM in early fibrotic lesions provides fibronectin and other adhesion molecules as ligands for regenerating epithelial cells to ensure successful repair [3,4,39-41]. In the murine bleomycin-induced pulmonary fibrosis model, we suggested that epimorphin in the early fibrotic lesions participated in adhesion of the regenerating alveolar epithelial cells. Involvement of epimorphin in the repair process was also reported for other organs including the gut during tissue repair in an isograft in rats [20] and the liver during regeneration after partial hepatectomy in mice [17]. Increased expression of epimorphin during epithelial cell regeneration was also reported in human skin ulcers and active ulcerative colitis [32,42].

We observed strong epimorphin immunostaining in early fibrotic areas with a few Î±-smooth muscle actin-positive cells in NSIP, as well as weak epimorphin immunostaining in fibroblastic foci with many Î±-smooth muscle actin-positive cells and desquamative regenerated alveolar epithelial cells in UIP. It is believed that in UIP, a failure of re-epithelialization of fibroblastic foci maintains fibroblast/myofibroblast activity and ECM synthesis [43,44]. We suggest that highly expressed epimorphin in the lungs of patients with NSIP may be necessary during wound healing, especially for regeneration of alveolar epithelial cells in early fibrotic lesions, and we also suggest that poorly expressed epimorphin in the lungs of patients with UIP may lead to failure of re-epithelialization of fibroblastic foci and the contraction of fibrotic lungs during the end stage of UIP.

### Role of Epimorphin Associated with MMP-2 in Lung Repair

To understand the role of epimorphin in the repair process in the lungs of patients with NSIP, we evaluated whether epimorphin enhanced expression of MMP-2 (gelatinase A). Recombinant epimorphin induced formation of some monolayer cell islands in addition to a loose cellular sheet and augmented expression of MMP-2 in cultured human alveolar epithelial cells, as demonstrated by immunohistochemistry and the MMP-2 Activity Assay System. We suggest that epimorphin-induced monolayer cell islands are a type of formation of differentiated alveolar epithelial cells, like the epimorphin-induced spheroid formation of rat hepatocytes, and suggest that the augmented expression of MMP-2 may be similar to the increased albumin production of rat hepatocytes.

We also demonstrated expression of MMP-2 in re-epithelialized cells overlying epimorphin-positive early fibrotic areas by means of double-labeled confocal fluorescent images. MMP-2, which is an important proteinase needed for matrix degradation, is secreted by type II alveolar epithelial cells *in vitro *[45]. Fukuda et al. [25] observed the localization of MMP-2 in fetal rabbit alveolar epithelial cells and gelatinolytic activities of MMP-2 in fetal rabbit lung, which indicated an important role for MMP-2 in formation of alveoli. These authors also observed the localization of MMP-2 in regenerating alveolar epithelial cells covering intra-alveolar fibrotic areas in a study of bronchiolitis obliterans organizing pneumonia, which is a reversible fibrotic human lung disease [13]. Yaguchi et al. [26] reported that MMP-2 was found in regenerating alveolar epithelial cells and that gelatinolytic activities of the active forms of MMP-2 increased in later repair stages in bleomycin-induced pulmonary fibrosis, during reconstruction of alveoli. Moreover, Fukuda et al. [46] reported that in bleomycin-induced pulmonary fibrosis, re-epithelialization on the surface of early intra-alveolar fibrotic lesions in gelatinase A^-/- ^mice was markedly reduced compared with that of gelatinase A^+/+ ^controls. Thus, MMP-2 was up-regulated and activated in regenerating alveolar epithelial cells, which may allow elongation and migration of these cells for successful repair of pulmonary fibrotic lesions [[26,27], 47].

We therefore suggest that epimorphin expression in early fibrotic lesions in the lungs of patients with NSIP may contribute to the repair process in pulmonary fibrosis, in part by inducing MMP-2 expression. What may follow this MMP-2 expression may be elongation and migration of regenerating epithelial cells and degradation of ECM in alveolar spaces. Thus, epimorphin, as a component of proteolytic systems, may contribute to cell migration and tissue remodeling during repair of human lung fibrosis.

Although epimorphin receptors have not yet been identified, binding ECM molecules has been suggested as involved in establishing epithelial polarity, thus helping form an organized basement membrane and cell-ECM junctional complexes [21]. Our results are consistent with the idea [22,34] that epimorphin has a high affinity for ECM molecules and could modify functions of adhesion and migration of regenerating alveolar epithelial cells by altering signaling through MMP-2.

In conclusion, epimorphin expressed in lungs may have important roles as a morphogen not only in mice but also in humans, and epimorphin may contribute to the repair process in human pulmonary fibrosis via epithelium-mesenchyme interactions.

## Supplementary Material

Additional File 1 Table 1. Characteristics of subjects, including pulmonary function test results Click here for file
