# Supplementary material for: Epimorphin expression in interstitial pneumonia
Source: Respir Res. 2005 Jan 16;6(1):6. doi: 10.1186/1465-9921-6-6 (PMC548284; doi:10.1186/1465-9921-6-6)
Supplement: Additional File 1 — Table 1. Characteristics of subjects, including pulmonary function test results [file 1465-9921-6-6-S1.doc]

| Variable | **UIP** | **NSIP** | **Control** |
| --- | --- | --- | --- |
| No. of subjects | 9 | 8 | 8 |
| Average age (yr) | 58.3±8.1 | 53.5±7.8 | 53.9±8.9 |
| No. of male/female | 6/3 | 3/5 | 4/4 |
| No. of nonsmokers/smokers | 5/4 | 5/3 | 3/5 |
| Duration of dyspnea (months) | 16.3±18.1 (0-120) | 14.4±11.1 (0-150) |  |
| FEV_1_, % predicted | 70.2±15.26 | 79.6±13.06 | 84.5±10.98 |
| FVC, % predicted | 68±4.66 | 78.4±4.83 | 83.6±4.06 |
| TLC, % predicted | 67.3±4.16 | 79.3±3.12 | 82±3.66 |
| Dl_co_, % predicted | 60±4.13 | 77±5.14 | 89±2.24 |
| CT findings | Reticular pattern, honeycombing, traction bronchiectasis | Ground-glass attenuation, irregular lines, consolidation |  |

Table 1

**Characteristics of subjects, including pulmonary function test results**
